# On the Treatment of Infantile Diarrhœa

**Published:** 1867-10

**Authors:** 


### On the Treatment cf Infantile Diarrhœa.

BY DR. BUIZ.

Dr. Buiz expresses the following opinions, as the results of his experience, on this subject:

"1.—The diarrhœa of spoon-fed infants generally yields to the addition of a small quantity of bicarbonate of soda or of lime-water to the milk.

"2.—In summer-diarrhœa supervening without any tangible cause, from one-sixth to one-quarter of a grain of calomel three or four times a day, associated with an equal amount of ipecacuanha, will often be found efficacious. If the indisposition is consequent on exposure to cold, minute doses of opium are appropriate.

"3.—Chronic diarrhœa resulting from various causes may in most cases be checked with nitrate of silver. one-sixth of a grain of which may be exhibited without risk. This remedy is sometimes, however, rejected by the stomach, and should then be replaced by tonics and vegetable astringents.

"4.—Diarrhœa combined with anæmia and impaired nutrition, is often the result of a state of decomposition of the blood, for which the best remedy is the proto-iodide of iron. In such cases bismuth is frequently unavailing, whereas in doses of half a drachm three times a day it is invariably successful against intestinal relaxations referable to tubercular ulceration. The causes of intestinal catarrh are, however, so obscure, that in many instances the treatment must be empirical."—*Journal of Practical Medicine & Surgery.*

